# Differences in stigmatizing attitudes towards people with mental illness between psychiatry trainees and Family Medicine trainees: A comparative study

**DOI:** 10.1192/j.eurpsy.2025.875

**Published:** 2025-08-26

**Authors:** M. K. Khamassi, B. N. Saguem, A. Rhouma, J. Nakhli

**Affiliations:** 1 Department of psychiatry, Farhat hached hospital; 2 Research Laboratory LR12ES04, University Of Sousse, Faculty Of Medecine Of Sousse, Sousse, Tunisia

## Abstract

**Introduction:**

Mental illness stigma continues to be a significant challenge in healthcare. Trainees in different medical fields may have varying levels of exposure and understanding, which can shape their attitudes towards patients with mental health conditions.

**Objectives:**

To examine differences in stigmatizing attitudes towards people with mental illness between Family Medicine trainees and psychiatry trainees.

**Methods:**

A comparative study was conducted. Psychiatry trainees affiliated with the faculties of medicine in Tunisia (n=120) and Family Medicine trainees affiliated with the faculty of medicine of Sousse (n=206) were invited to respond to a survey comprising the Attribution Questionnaire (AQ-27), a measure that evaluates nine stigma factors, blame, pity, anger, help, dangerousness, fear, segregation, avoidance, and coercion. Higher scores indicated more endorsed stigma. Self-report measures of affirming attitudes were also used, including the Self-Determination Scale (SDS), the Empowerment Scale (ES), and the Recovery Scale (RS). Higher scores represent enhanced views of these concepts.

**Results:**

In total, 94 psychiatry trainees and 66 Family Medicine trainees responded to the survey, with respective response rates of 78% and 32%. The two groups were comparable in terms of age, gender, family and personal psychiatric histories.

Family Medicine trainees reported significantly higher AQ-27 total scores (p=.042). Additionally, they reported significantly higher scores for blame (p=.025), dangerousness (p=.006), fear (p=.048), and segregation (p=.005) stigma factors.

No significant differences between the two samples were found in avoidance (p=.525), coercion (p=.379), pity (p=.741) and help (p=.092).

Concerning affirming attitudes, there were no significant differences between the two groups in SDS (p=.148), RS (p=.552), and ES (p=.727) scores.

**Conclusions:**

Results revealed that psychiatry trainees endorse less stigmatizing attitudes towards patients with mental illness, particularly regarding the dangerousness of these patients. Nevertheless, they still endorse negative attitudes regarding the concept of recovery and affirming attitudes towards patients with mental illness. Anti-stigma interventions should promote not only increased contact but also other strategies that will promote believing in recovery and social inclusion.

**Disclosure of Interest:**

None Declared

